# Effect of parent attendance at an adult commercial weight management programme on their children

**DOI:** 10.1136/bmjpo-2025-004417

**Published:** 2026-06-03

**Authors:** Ruth Mears, Deborah Sharp, Aidan Searle, Ruth Salway, Julian Hamilton-Shield

**Affiliations:** 1 Centre for Academic Primary Care, University of Bristol, Bristol, UK; 2 Population Health Sciences, Bristol Medical School, University of Bristol, Bristol, UK; 3 NIHR Bristol Biomedical Research Centre, University Hospitals Bristol and Weston NHS Foundation Trust and University of Bristol, Bristol, UK

**Keywords:** Obesity, Child Health, Health Policy, Qualitative research

## Abstract

**Objectives:**

This study aimed to explore what motivates parents to address their own weight and support their child to reach or maintain a healthy weight, how parents assess their child’s weight and the impact of a parent addressing their own weight on their child.

**Methods:**

In this mixed-methods study, Slimming World members (a UK-based adult commercial weight management programme) who had children aged 5–11 years old were invited to complete an online survey and telephone interview. This included parent-reported measures of Body Mass Index (BMI) and their child’s weight status, physical activity and dietary behaviours. Quantitative survey data were summarised descriptively using percentages to identify associations of both parental weight management motivating factors and child weight concerns with parent weight category. Qualitative telephone interview analyses followed an inductive thematic approach.

**Results:**

Improving health (81%) and dissatisfaction with appearance (81%) were the most common motivators for parents to address their own weight. 67% of parents who were worried about their child’s weight felt more motivated to help their child reach a healthier weight since they had taken steps to address their own weight. Weight-related adverse experiences during the parents’ lives were an important driver to address their child’s weight, as were health concerns. Parents assessed their child’s weight mainly through clothing size and visual comparison with peers rather than weighing. Concern about a child’s weight increased with parental BMI and child age. Since the parents addressed their own weight, 48% reported their child’s diet was healthier, and 27% that their child was more physically active.

**Conclusion:**

Parents addressing their own weight are particularly motivated to help their child reach or maintain a healthy weight. Targeted, child-oriented interventions at this time may enhance the reported indirect benefits on children.

**WHAT IS ALREADY KNOWN ON THIS TOPIC**

**WHAT THIS STUDY ADDS**

- Parents addressing their weight through lifestyle weight management support reported improvements in their child’s diet (48%) and physical activity levels (27%), as well as increased motivation to support their child reach a healthier weight. Key reasons for dietary improvements included changes in family meal preparation and a general healthier emphasis within the household.

**HOW THIS STUDY MIGHT AFFECT RESEARCH, PRACTICE OR POLICY**

- Offering or sign-posting to child weight management support when a parent engages in a lifestyle weight management programme could provide a valuable opportunity to augment any positive indirect effects on the child and the opportunity to take objective measures of possible adiposity.
- Future research should examine whether beneficial collateral effects on children are observed when parents adopt a predominantly pharmacological approach to managing their weight.

## Introduction

 Globally, the prevalence of obesity has nearly tripled between 1975 and 2016.^1^ In England (2019), 64% of adults and 30% of children were affected by overweight or obesity.^2^ Children are at increased risk of developing obesity if a parent is living with overweight or obesity.^3^ Obesity has been shown to track from childhood into adulthood, with associated health and socioeconomic consequences.^4^,^7^ Breaking the cycle of intergenerational obesity is therefore important for reducing obesity prevalence.^8^

Obesity is complex and multifactorial, with an important aspect of management focused on achieving a healthy balance between energy intake and expenditure. This requires behavioural change to modify both dietary intake and increase physical activity levels or reduce sedentary behaviour.^9^ Behavioural change, in turn, requires an individual to be motivated to effect change, so understanding motivational factors can help ensure support is offered at an opportune time point and intervention design tailored to the individual.

In the UK, adults may decide to address their weight by joining Slimming World (SW). SW is the largest UK-based adult commercial weight management programme, offering in-person group and online support. The time point when a parent addresses their own weight may represent an opportune moment to consider the needs of children residing in the same household, in terms of reaching or maintaining a healthy weight. Parent role modelling of healthy dietary and physical activity behaviours has been shown to be positively associated with a child’s healthy lifestyle behaviours.^10 11^ Furthermore, there is increasing evidence supporting ‘parents as agents of change’ in helping children reach a healthier weight,^12^ with a lack of parental engagement being a well-recognised barrier to a child accessing appropriate support.

Despite the known parent–child association in weight status,^13 14^ few studies have explored the effects on a child when their parent joins an adult weight management programme. With the recent emergence of weight management drugs for adults, such as Semaglutide and Tirzepatide, there is an increasing shift towards adults adopting a pharmacological approach to addressing their weight. However, these medications focus on the individual and may not be accompanied by behavioural change interventions, which may have wider-reaching benefits to the family. Therefore, exploring the collateral effects of adult weight management programmes, without pharmacological support, is important to understand the benefits of such an approach beyond the parents themselves.

This study focused on SW members who were parents of children aged 5–11 years old. The study aimed to explore (1) what motivates parents to address their own weight and to help their child reach or maintain a healthy weight, (2) parents’ perception and worries about their child’s weight and (3) the collateral effect on a child (if any), when a parent decides to address their own weight.

## Methods

### Participants

SW members who were parents of children aged 5–11 years old were eligible to take part in the online survey, regardless of whether they were worried about their child’s weight. The participant cohort is described elsewhere in a separate analysis focused on exploring the feasibility of engaging parents attending an adult weight management programme with child weight management support.^15^ Recruitment to the survey was through a link on the members-only section of the SW website. The survey link was also distributed to SW Consultants in their weekly email communication from Head Office, to share with SW members.

Telephone interview participants were sampled from the respondents of the online survey and a recruitment advert on the members-only section of the SW website. Parents were eligible to take part in the telephone interviews if they were worried that their child, aged 5–11 years old, was living with overweight or obesity.

### Study design

This was a mixed-methods study including descriptive quantitative analyses of a cross-sectional survey and qualitative analyses of telephone interview data and free-text survey responses. The survey was developed on the ‘Online Surveys’ platform (https://www.onlinesurveys.ac.uk/). Multiple choice (single answer) questions collected data related to the parent’s sex, age group, height and weight on joining SW (self-reported), length of SW membership, age of children in their household (for children aged 5–11 years old), whether the parent was worried about their child’s weight and how motivated parents felt to help their child reach or maintain a healthy weight. Multiple choice (multiple response) questions collected data on the parents’ motivations to address their own weight and the collateral effect (if any) of the parents addressing their own weight on their child (parent-reported). Free text questions collected the parents’ postcode (to calculate the Index of Multiple Deprivation (IMD) score) and the reasons why they felt motivated to help their child reach or maintain a healthy weight.

The telephone interview topic guide was informed by findings from the online survey and developed by RM, DS and JHS. All interviews were conducted by RM (a primary care clinical research fellow) via telephone at a time and place convenient for the participant. Interviews were audio-recorded and transcribed, with identifiable information anonymised. Participants were not known to the researchers prior to study commencement.

### Analysis

Socioeconomic deprivation was reported for English residents using their reported postcode, according to the English IMD score 2019.^16^ Self-reported parental Body Mass Index (BMI) data were categorised according to National Institute for Health and Care Excellence (NICE) classification as healthy weight (BMI 18.5–24.9 kg/m^2^), overweight (BMI 25–29.9 kg/m^2^), obesity class 1 (BMI 30–34.9 kg/m^2^), obesity class 2 (BMI 35–39.9 kg/m^2^) and obesity class 3 (BMI of 40 kg/m^2^ or more).

Quantitative survey data were analysed in Microsoft Excel and Stata V.18.0,^17^ while free text survey responses were analysed in N-vivo through content analysis. Due to the nature of the survey, the quantitative analysis was primarily descriptive. Survey data summarising motivating factors for the parent to address their own weight were presented as cross-tabulations by the parent’s weight category (overweight, obesity 1, obesity 2 or obesity 3). Survey data related to parental concern about a child’s weight were also presented as cross-tabulations by the parents’ weight category, IMD quintile and length of SW membership. To aid interpretation, we reported p values for χ^2^ tests of independence to identify which patterns may be of potential future interest, and which are attributable to random variation. However, these should be treated as indicative only, to avoid unwarranted attention on the latter, rather than as formal hypothesis tests.

Telephone interview data were analysed through an inductive thematic approach, using N-vivo 1.6.1 software, by RM and AS (an experienced researcher in qualitative methodology and health services research).^18^ This approach was considered appropriate to maximise exploration of the ideas and concepts emerging within the telephone interviews. After the analysts had independently familiarised themselves with the data, they proceeded to open coding, meeting to agree on a coding frame. Initial themes were developed through identifying patterns of shared meaning for the analysts. The themes identified helped contextualise aspects of the survey data.

### Patient and public involvement

Patients or the public were not involved in the design, conduct, reporting or dissemination plans of our research.

## Results

### Participant characteristics

The survey was completed by 396 SW members (98% female). 18 telephone interviews were conducted. Participant characteristics are published elsewhere.^15^ The average interview length was 43 min (range 24–80 min). 13 interview participants had taken part in the online survey. Five interview participants were recruited via a recruitment advert on the SW website. Further participant characteristics are detailed in online supplemental table 1). The age of the children in the households was equally distributed among 5–11-years-olds. There was a similar proportion of participants representing each IMD quintile. The survey was conducted between August and October 2020, and the interviews between March and November 2021.

### Parental motivation to address their own weight

From online survey data, the most common motivators for parents to address their own weight were to improve their health (81%) and because they were unhappy with how they looked (81%) (table 1). The higher the parental BMI, the greater the motivation to be a healthier role model for their child (overweight: 56%, obesity 1: 66%, obesity 2: 72% and obesity 3: 81%) and to improve their own health (overweight: 64%, obesity 1: 81%, obesity 2: 84%, obesity 3: 92% and table 1).

**Table 1:** Motivation for parent to address their own weight

Motivating factor	All parents (n=395*) (%)	Parental weight category*				
		Overweight (n=77) (%)	Obesity 1 (n=112) (%)	Obesity 2 (n=98) (%)	Obesity 3 (n=91) (%)	P value†
To improve health	81	64	81	84	92	<0.001
Unhappy with how I looked	81	79	85	79	82	0.639
To feel better about myself	80	70	85	79	80	0.109
To be a healthier role model for my children	67	56	66	72	81	0.003
To feel fitter	58	52	54	64	62	0.249
To fit into certain clothes	44	48	39	46	43	0.636
For a specific occasion (eg, a wedding)	12	9	10	15	16	0.319
To support a family member (adult) with their weight loss	7	8	7	10	3	0.326
To support a friend with their weight loss	5	8	4	7	3	0.389
Referred by a health professional	3	1	3	2	4	0.623
To support a family member (child) achieve a healthier weight	3	4	2	0	8	0.018
Other	4	0	4	4	5	NA

Data source: online survey, multiple response and multiple choice question.

* One parent (n=1/396) did not provide a BMI. 17 parents (n=17/396) were within a healthy BMI category, and their motivations are not presented here.

† P values for χ2 tests of independence are presented to aid interpretation only, and should not be interpreted as formal hypothesis tests.

BMI, Body Mass Index.

### Parental motivation to address their child’s weight

Since taking steps to address their own weight, 67% of parents with concerns about their child’s weight felt more motivated to help their child reach a healthier weight. A further 26% of parents stated that they have always felt motivated. Thus, overall, 93% of parents concerned that their child was living with overweight or obesity felt motivated to help their child reach a healthier weight.

From qualitative analysis of free-text survey responses, the perceived reasons why parents felt motivated included (1) parents’ past experiences and wanting to protect their child, (2) current and future health, (3) psychosocial factors and (4) to help the child develop a healthy lifestyle to continue into adulthood (table 2).

**Table 2:** Motivations for parent to help their child reach or maintain a healthy weight

Theme	Subtheme	Quotes
Parents’ past experiences (including weight-related stigmatisation) and wanting to protect their child	I don’t want them to go through what I did	*‘*I was a very large child. School was very hard for me and for that reason I do everything I can to ensure my children don’t have to go through the same.’ (N205, female 26–35 years) ‘It upsets me she has the same feelings of herself that I once did. No child should have those emotions.’ (N22, female 26–35 years) ‘I never want my child to experience the emotional and mental pain of dieting/bingeing and an unhealthy relationship with food like I have.’ (N283, female 36–45 years)
	I don’t want them to end up like me	*‘*After dealing with obesity for most of my life I fear my daughter will end up like me.’ (N212, female 36–45 years) ‘Not wanting her to end up like me hating herself and looking disgusting, no clothes fitting, etc’ (N253, female 36–45 years)
	I don’t want them to feel like I do	*‘*I don’t want her to feel like I do, embarrassed and unfit’ (N346, female 46–55 years) ‘I wouldn’t want my child to feel how I feel about myself about themselves.’ (N50, female 26–35 years)
Current and future health	For current and/or future ‘good’ health	*‘*To show him how to be healthy and fit to take into adulthood’ (N337, female 36–45 years) ‘Their short and long-term health’ (N58, female 46–55 years)
	To protect against health-related complications of obesity	*‘*To reduce risk of serious health problems later in life’ (N51, female 36–45 years) ‘Do not want them to have any health problems related to weight.’ (N102, female 36–45 years)
Psychosocial factors	To be ‘happy and healthy’	*‘*For them to have a healthy happy life without worry’ (N358, female 36–45 years)
	For confidence and self-esteem	*‘*She’s body conscious and school kids can be so mean. I want her to love herself before any other feelings shadow that.’ (N213, female 20–25 years) ‘Allowing her to be confident in herself and have a positive body image.’ (N196, female 36–45 years)
	To ‘fit in’ with peers and prevent or stop bullying	*‘*I do not want my child to be bullied by others at school for being the fat kid.’ (N342, female 36–45 years)
To develop a healthy lifestyle as a child to continue into adulthood	To develop healthy habits for life	*‘*I know it is easier to make it a habit for her now rather than something to learn later.’ (N61, female 46–55 years) ‘Letting them see that if they take the right steps now and learn about nutrition and eating a healthy balanced diet they can have a healthy future.’ (N361, female 26–35 years)
	To develop a healthy relationship with food	*‘*I want to set them up with a healthy relationship with food.’ (N156, female 36–45 years)
	To keep physically active	‘I want him to be healthy and enjoy physical activity’ (N106, female 36–45 years)

Data source: online survey, free text answer.

### Parental assessment of the child’s weight status

Two main themes emerged from the telephone interviews about how parents assess a child’s weight:

#### Clothes size

Most parents referred to clothes sizes when discussing how they worked out whether their child was a healthy weight or not.

I’d had to start buying him the plus sized school clothes which I was quite shocked about, so I would say within the last couple of years I’ve noticed that he has needed bigger clothes than probably the average child*.* (Interview 14, female parent, 36–40 years)

#### Visual assessments

Many parents described making visual assessments of their child’s weight, with some making weight comparisons with other children. Few parents weighed their child.

I just look at them. I don’t ever weigh them, I don’t ever do anything, I just look at them. (Interview 1, female parent, aged 46–50 years)

I suppose I look at them compared with their peers if I’m honest. I’ll look at my boy and my daughter and I see that they’re the biggest in the class*.* (Interview 11, female parent, 36–40 years)

Survey data identified that the proportion of parents concerned about one or more of their children’s weights increased as the BMI category of the parent increased (overweight: 25%, obesity 1: 28%, obesity 2: 32% and obesity 3: 51%) (table 3). There was no association between parental worry about a child’s weight and IMD quintile or SW membership length.

**Table 3:** Characteristics associated with parental concern about a child’s weight

		Proportion of parents worried ≥1 child is living with overweight or obesity (%)	P value*
Parental BMI on joining SW†	Overweight	25	
	Obesity 1	28	
	Obesity 2	32	
	Obesity 3	51	
			0.001
IMD quintile‡	1 most deprived	38	
	2	38	
	3	30	
	4	36	
	5 least deprived	32	
			0.826
Length of SW membership	0–12 months	33	
	>12–24 months	32	
	>24–36 months	40	
	>36 months	34	
			0.800

Source: online survey data, n=396.

* P values for χ2 tests of independence are presented to aid interpretation only, and should not be interpreted as formal hypothesis tests.

† One parent (n=1/396) did not provide weight/height data. 17 parents were within a healthy weight category, and their data are not presented here.

‡ IMD quintile reported only for survey participants residing in England (n=338/396).

BMI, Body Mass Index; IMD, Index of Multiple Deprivation; SW, Slimming World.

**Figure 1:**
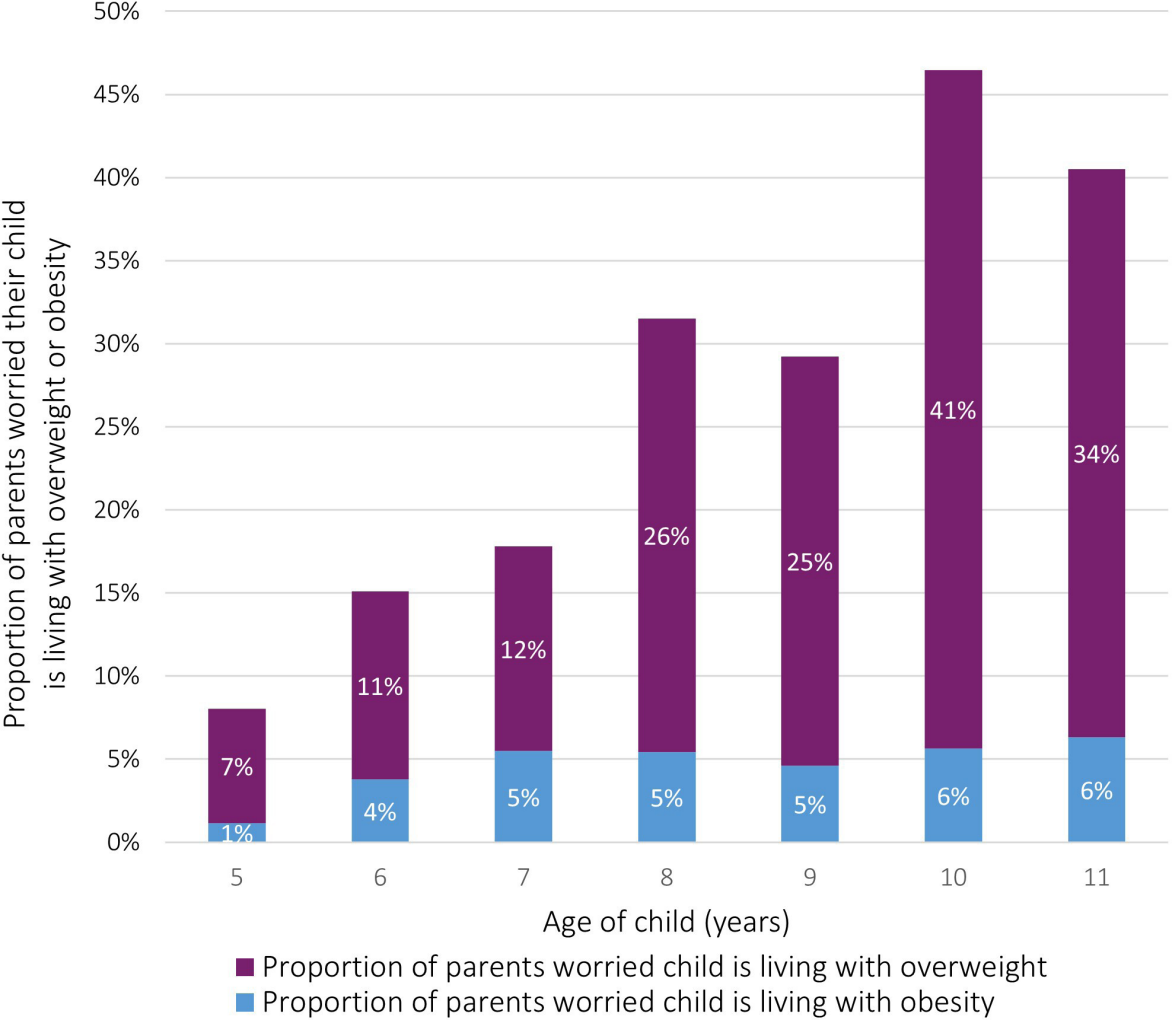
Parental concern about the child’s weight according to the age of the child.

Parents of older children were more likely to worry that their child was living with overweight or obesity than parents of younger children (41% of parents of 11-year-olds vs 8% of parents of 5-year-olds, figure 1).

### Effect of parents addressing their own weight on the child’s diet

From the online survey data, nearly half of parents (48%) reported that one or more of their children had a healthier diet, since the parent had taken steps to address their own weight (table 4). The telephone interviews explored this further, with two main themes emerging: (1) family meal preparation and (2) a general healthier emphasis in the household.

**Table 4:** Collateral effect of parents addressing their weight on their child’s diet, physical activity levels and weight

	All parents (n=396) (%)	Parent worried that ≥1 child is living with overweight or obesity (n=134) (%)	Parent not worried that any children are living with overweight or obesity (n=262) (%)
Child has a healthier diet	48	51	47
Child does more physical activity	27	31	24
Child’s weight has improved	6	12	3
No changes to the child	46	42	48
Unsure	6	6	5
No changes made by the parent yet	5	10	3

Source: online survey data, multiple-choice, multiple-response question.

For parents with multiple children aged 5–11 years, the data presented in this table represent instances where a change was observed in one or more of their children.

#### Family meal preparation

Many parents switched to lower-fat options, such as lower-fat meats, and these changes were implemented across the family. Some parents also described changing the way they cooked for their family, avoiding lots of oils and using alternatives, for example, sprays/air-fryers.

I go for lean beef, I go for lean pork loin and I cut all the fat off. So, my meats have all changed. I don’t buy any fatty meats. (Interview 1, female, age 46–50 years)

I suppose I did change for the family because I stopped frying in the oil and I’d fry in Fry Light. Now I can’t even go back… (Interview 6, female parent, 46–50 years)

Other changes shared among the family included switching from white to wholemeal bread and reducing sugar intake.

I’d change my shopping so I’d buy wholemeal bread instead of white, so I suppose my family was introduced to it but without me even knowing. *(*Interview 6, female parent, 46–50 years)

#### General healthier emphasis in the household

Some parents described a more general emphasis on healthier foods and snacking. A few reported the family had fewer takeaways than previously.

I suppose the things that we have in the house are generally more geared towards a healthy lifestyle. (Interview 4, female parent, 41–45 years)

When I’m on Slimming World we all eat better because in my house, Fridays is takeaway night and it’s set in stone and when I’m on Slimming World nobody else gets one ‘cos I’m not, so I cook instead. (Interview 16, female parent, age 26–35 years)

### Effect of parents addressing their own weight on children’s physical activity levels and weight

The online survey data indicated that physical activity levels in the child (parent-reported) were less impacted by the parent taking steps to address their own weight, with only 27% of parents stating that one or more of their children did more physical activity (table 4). 12% of parents who were worried about their child’s weight felt there had been an improvement in one or more of their children’s weight since they had taken steps to address their own weight. 46% of parents did not observe any changes in their children’s diet, physical activity or weight.

## Discussion

### Main findings

Nearly all parents surveyed (98%) were female, and all parents interviewed were female. Survey data showed that parents attending SW were motivated to address their own weight, for health reasons, but also due to being unhappy with their appearance. As parental BMI classification increased, the proportion of parents motivated by a desire to improve their health or to be a healthier role model for their child also increased.

Parents of older children were more likely to be concerned about their child’s weight than those of younger children. Nearly all parents surveyed who were worried that their child was living with overweight or obesity felt motivated to help their child reach a healthier weight (93%). Around two-thirds felt more motivated, since they had taken steps to address their own weight. Child-related adverse events, such as stigmatisation during the parents’ early life, were one of the main drivers for wanting to help their child, alongside health concerns. Nearly half of parents reported collateral benefits for one or more of their children regarding a healthier dietary intake, since they had taken steps to address their own weight. Just under a third of parents reported that one or more of their children were more physically active. A minority of parents reported an improvement in one or more of their children’s weight status.

### Contextualisation and implications of findings

Adults commonly report health or appearance concerns as motivations for seeking to improve their own weight.^19^ Our study found parents were frequently motivated to help their child reach or maintain a healthier weight, due to their own adverse early life and adulthood experiences, including weight-related stigmatisation and psychological impacts of living with overweight or obesity. For adults, weight stigmatisation has been shown to contribute to increased weight gain over time, rather than weight loss.^20^ The trauma some parents have experienced due to their weight warrants clear acknowledgement in family-based weight management programmes, due to the complexities of weight stigma related to the parent–child dyad.^21^

In our study, parents described assessing their child’s weight status through visual assessment and clothes size, rather than direct measurements. This tendency towards subjective assessments by parents of child weight status has also been reported in other studies.^22 23^

Only 8%–15% of parents with a child aged 4–5 years old were worried that their child was living with overweight or obesity in our cohort. This is notably lower than the prevalence (23%) reported by the National Child Measurement Programme (NCMP) for this age group.^24^ In contrast, 41%–46% of parents with a child aged 10–11 years old were worried that their child was living with overweight or obesity, which is higher than the prevalence reported by NCMP (35%).^24^ It is unlikely that the true prevalence of overweight or obesity in 4–5-year-olds within our study is lower than the NCMP data. Instead, this finding supports other studies in the literature suggesting that parents of younger children are more likely to underestimate their child’s weight status than parents of older children.^25^,^27^ The higher proportion of parents worried about their child aged 10–11 years old living with overweight or obesity likely also reflects the known higher prevalence of overweight and obesity in this age group, combined with the heritable risk.^3^

Parental acknowledgement of their child’s adiposity status is key to engagement with childhood weight management support. The high proportion of SW members reporting concern suggests this group might be a suitable target for referral onwards, with a better chance of engagement.

The higher the parental BMI, the more likely the parent was to report concern about their child’s weight. This is in line with findings from other studies, reporting that higher parental BMI to be associated with higher children’s BMI.^28^ Parents with a higher BMI were also more likely to be motivated by a desire to be a healthier role model for their child. Parental role-modelling has been reported as an effective strategy to improve physical activity and healthy dietary intake.^10 29^ Therefore, the combination of children at higher risk of developing obesity, with motivated parents’ role-modelling healthy lifestyle changes, again suggests this would be an opportune moment for adult weight management programmes to signpost families directly to child weight management support. Families in which the parental BMI was highest may have potential to benefit from this the most. This is further supported by the strong evidence base for parents and children making lifestyle changes together,^30^ despite the current lack of a routine referral pathway between adult and child weight management programmes.

There is limited literature examining the collateral impact on children when parents take steps to address their own weight through accessing adult weight management support.^31^ One of the few studies examining the effect of an adult commercial weight management programme on their offspring was limited by its small sample size (n=20 parents) as a feasibility study and only found a beneficial effect in terms of a reduction in saturated fat consumption.^32^ Brown *et al*^33^ reported utilisation of weight control practices (both healthy and unhealthy) in children, where parents participated in an adult weight management programme.^33^ For adults undergoing bariatric surgery, positive collateral effects for children within the same household have been noted when comparing presurgery and postsurgery measures, in terms of physical activity, reduced screen-time and a child’s weight status lower than expected.^34^ Our study demonstrates the potential beneficial effects for some children, not just in terms of diet and physical activity, but also related to improved parental motivation to effect change for their child. The collateral effects reported by some parents on their children from lifestyle adult weight management programmes warrant further exploration with objective measures. This is particularly important given the rising popularity of pharmacological obesity treatments, which we hypothesise may offer limited indirect benefits to children if given in isolation, without accompanying behavioural change interventions.

### Limitations

Most parents included in this study were female, therefore paternal perspectives are not fully represented. However, as most adults accessing behavioural adult weight management programmes in the UK are female, the findings of this study have reasonable generalisability for this cohort of interest.^35 36^ Recruitment into the study was via a single adult weight management provider, which may introduce sampling bias. However, SW is the largest commercial provider in the UK and is accessed both through National Health Service referral pathways and on a fee-paying basis, increasing the likelihood that the sample reflects a broad range of socioeconomic backgrounds. Regarding reflexivity in the qualitative aspects of the study, it is recognised that RM’s experience both as a general practitioner and parent had the potential to influence data collection, but open-ended questions helped mitigate this. Furthermore, the secondary male data analyst (AS) did not have a prior interest in the research topic other than that of a methodologist and served to counter the potential for implicit bias in the analysis of interview data.

Parental BMI data relied on self-reported weight and height, which although has some validity,^37 38^ are less accurate than directly measured height and weight. The child’s weight status was not independently measured. However, as parents typically underestimate their child’s weight status, it is reasonable to assume that, in most cases, a parent’s concern regarding their child’s weight was justified.^25^ Dietary and physical activity changes also relied on self-report data from parents, which may be subject to recall bias. As weight improvement often lags after dietary and physical activity changes, it is possible that any collateral impact on a child’s weight may not have been evident at the time of parental report. Data collection occurred during the COVID-19 pandemic, and associated confounding factors may have influenced parents’ responses. As the survey was advertised on the SW members-only section of their website, it is not possible to report a response rate.

While in the survey, the focus was on whether there were any collateral benefits rather than negatives to a child’s nutrition, physical activity and weight, the telephone interviews included open-ended questions at which time parents could have brought up any perceived negative effects on their child. No such themes emerged. Finally, the study focuses on parental perspectives only, given that NICE advises families or carers to take the main responsibility for behavioural changes in children and young people, especially children under 12.^9^ However, further studies may wish to explore the child’s perspective.

## Conclusion

Most parents actively taking steps to address their own weight were motivated to help their child reach a healthier weight, and some reported that their child’s diet and physical activity levels had improved. Future research should explore these subjective parental observations using objective measures. Parents often described a lifelong battle with their weight, and acknowledgement of the complex interplay between parents’ early life negative experiences, and their desire to protect their child from this, warrants consideration within family-based weight management programmes. Further research to explore the potential of routinely linking adult weight management services to child weight management programmes may provide an opportunity to help families achieve a healthier weight together, at a time point in their lives where the adult has already decided to engage.

## Supplementary material

10.1136/bmjpo-2025-004417 online supplemental file 1

## Data Availability

Data are available upon reasonable request.
