# Supplementary material for: Effect of parent attendance at an adult commercial weight management programme on their children
Source: BMJ Paediatr Open. 2026 Jun 3;10(1):e004417. doi: 10.1136/bmjpo-2025-004417 (PMC13239348; doi:10.1136/bmjpo-2025-004417)
Supplement: online supplemental file 1 [file bmjpo-10-1-s001.docx]

### **Supplementary Table 1**: Participant characteristics

|  | | Online survey participants | | Telephone interview participants | |
| --- | --- | --- | --- | --- | --- |
|  |  | Number of participants (n) | % or mean (SD) | Number of participants (n) | % or mean (SD) |
| Sex of parent | Female | 388 | 98% | 18 | 100% |
|  | Male | 8 | 2% | 0 | 0 |
|  | *Total* | *396* |  | *18* |  |
| BMI of parent on  joining Slimming World* | Healthy weight | 17 | 4% | 0 | 0% |
|  | Overweight | 77 | 19% | 2 | 11% |
|  | Obesity 1 | 112 | 28% | 5 | 28% |
|  | Obesity 2 | 98 | 25% | 6 | 33% |
|  | Obesity 3 | 91 | 23% | 5 | 28% |
|  | *Total* | *395* | *35.2 (6.8)* | *18* | *36.4 (5.4)* |
| Age of parent (years) | ≤25 | 4 | 1% | 0 | 0% |
|  | 26-35 | 111 | 28% | 4 | 22% |
|  | 36-45 | 195 | 49% | 8 | 44% |
|  | 46-55 | 80 | 20% | 5 | 28% |
|  | >55 | 6 | 2% | 1 | 6% |
|  | *Total* | *396* |  | *18* |  |
| Age of children in household (years) ** | 5 | 87 | 17% | 2 | 7% |
|  | 6 | 53 | 10% | 3 | 10% |
|  | 7 | 73 | 14% | 3 | 10% |
|  | 8 | 92 | 18% | 5 | 17% |
|  | 9 | 65 | 13% | 4 | 14% |
|  | 10 | 71 | 14% | 4 | 14% |
|  | 11 | 79 | 15% | 5 | 17% |
|  | 12 | NA | NA | 3 | 10% |
|  | *Total* | *520* |  | *29* |  |
| IMD Quintile *** | 1 *Most deprived* | 35 | 18% | 4 | 24% |
|  | 2 | 26 | 20% | 5 | 29% |
|  | 3 | 36 | 23% | 1 | 29% |
|  | 4 | 30 | 20% | 2 | 6% |
|  | 5 *Least deprived* | 43 | 19% | 1 | 12% |
|  | *Total* | *338* |  | 17 |  |
| **Healthy weight, BMI ≥18.5 to 24.9; Overweight, BMI ≥25 to 29.9; Obesity 1, BMI 30 to 34.9; Obesity 2, BMI ≥35 to 39.9; Obesity 3, BMI ≥40.*  ***Some participants recruited from the online survey, may have children who turned 12 by the telephone interview date. Parents participating in the survey may have ≥ 1 child in their household.*  **** IMD Quintile is only reported for survey participants residing in England.* | | | | | |
