# Supplementary material for: Effect of parent attendance at an adult commercial weight management programme on their children
Source: BMJ Paediatr Open. 2026 Jun 3;10(1):e004417. doi: 10.1136/bmjpo-2025-004417 (PMC13239348; doi:10.1136/bmjpo-2025-004417)
Supplement: Supplementary file 2 [file bmjpo-2025-004417.reviewer_comments.pdf]

PEER REVIEW HISTORY

BMJ Paediatrics Open publishes all reviews undertaken for accepted manuscripts. Reviewers are asked to complete a checklist review form and are provided with free text boxes to elaborate on their assessment. These free text comments are reproduced below.

ARTICLE DETAILS

**TITLE (PROVISIONAL)**	Effect of parent attendance at an adult commercial weight management programme on their children
**AUTHORS**	Mears, Ruth<br>sharp, deborah<br>Searle, Aidan<br>Salway, Ruth<br>Hamilton-Shield, Julian

VERSION 1 – REVIEW

**REVIEWER NAME**	sakamuri, Kavitha
**REVIEWER AFFILIATION**	
**REVIEWER CONFLICT OF INTEREST**	No Conflict of Interest
**AI DISCLOSURE**	No, I have not used AI
**DATE REVIEW RETURNED**	05-Feb-2026

**GENERAL COMMENTS**	With respect to terminology ,it is important that the manuscript uses the phrase children living with overweight / obesity consistently instead of the phrase children with overweight/ obesity.<br><br>With respect to the data interpretation regarding child's weight improvement, the mention of a twelve percent success rate must be considered in the context of a lagging indicator. It must be emphasized that actual improvement in weight may take more time to achieve than the immediate improvements in dietary practices or physical activity. This must be highlighted as an important point of consideration for practitioners to be aware of in order to properly manage the expectations of parents.<br><br>Parents used subjective methods such as clothing size comparisons or visual assessments, they may have unintentionally underestimated their child’s weight. It is therefore recommended that professionals consider objective weight measurement, using weighing scales data, especially for parents of young children.

**REVIEWER NAME**	Das, Sarthak
**REVIEWER AFFILIATION**	All India Institute of Medical Sciences - Deoghar, Pediatrics
**REVIEWER CONFLICT OF INTEREST**	No Conflict of Interest
**AI DISCLOSURE**	No, I have not used AI
**DATE REVIEW RETURNED**	13-Feb-2026

**GENERAL COMMENTS**	COMMENTS (Conceptual & Methodological)<br>•Clarify the mixed-methods design and explicitly describe how

	quantitative and qualitative findings were integrated.<br>•Acknowledge self-selection bias and limited generalisability due to recruitment through Slimming World and the predominantly female sample.<br>•Highlight the absence of objective child outcome measures and reliance on parent-reported diet, physical activity, and weight changes.<br>•Report effect sizes and consider multivariable regression analyses; address multiple testing concerns.<br>•Clarify the cross-sectional nature of the study and potential confounding due to the COVID-19 period.<br>•Reframe pharmacological comparisons (e.g., GLP-1 agonists) as hypothesis-generating rather than evidence-based conclusions.<br>COMMENTS (Interpretation & Depth)<br>•Avoid overinterpretation of collateral benefits and emphasise heterogeneity of observed effects.<br>•Include a brief gender-focused discussion highlighting the absence of paternal perspectives.<br>•Explicitly acknowledge the absence of the child's voice as a limitation.<br>•Expand interpretation of IMD findings and socioeconomic implications.<br>QUALITATIVE METHODOLOGY<br>•Add a reflexivity statement describing the interviewer's background and potential influence on data collection.<br>•Strengthen reporting of qualitative rigour by describing double coding, audit trail, and adherence to COREQ standards.<br>PRESENTATION & STRUCTURE<br>•Simplify dense tables and highlight statistically meaningful findings more clearly.<br>•Improve narrative explanation of figures within the Results section.<br>•Refine language to remove minor grammatical inconsistencies and improve conciseness.<br>ETHICAL & CONCEPTUAL CONSIDERATIONS<br>•Ensure consistent use of person-first and non-stigmatising language throughout the manuscript.<br>•Acknowledge potential unintended negative collateral effects, such as transmission of dieting behaviours or food restriction to children.<br>KEY RECOMMENDATIONS BEFORE PUBLICATION<br>•Strengthen the limitations section, particularly addressing bias, self-reporting, and lack of causal inference.<br>•Temper conclusions regarding collateral benefits.<br>•Enhance qualitative methodological transparency.<br>•Reduce speculative discussion not directly supported by data.<br>•Consider additional adjusted statistical analyses if data permit.

VERSION 1 – AUTHOR RESPONSE

Reviewer 1 - Dr. Kavitha Sakamuri

Comment 1: With respect to terminology, it is important that the manuscript uses the phrase children living with overweight / obesity consistently instead of the phrase children with overweight/ obesity.
Response 1: We have adjusted terminology, editing 'children with overweight/obesity' to 'children living with overweight or obesity' in the 'limitations' section and ''if their child, aged 5-11 years old, was overweight' to 'if their child, aged 5-11 years old, was living with overweight or obesity' in the results section. We have also edited 'children at higher risk of obesity' to 'children at higher risk of developing

obesity' in the discussion.

Comment 2: With respect to the data interpretation regarding child's weight improvement, the mention of a twelve percent success rate must be considered in the context of a lagging indicator. It must be emphasized that actual improvement in weight may take more time to achieve than the immediate improvements in dietary practices or physical activity. This must be highlighted as an important point of consideration for practitioners to be aware of in order to properly manage the expectations of parents.
Response 2: We have added a sentence to the limitations (in bold) as follows: 'Dietary and physical activity changes also relied on self-report data from parents, which may be subject to recall bias. 'As weight improvement often lags after dietary and physical activity changes, it is possible that any collateral impact on a child's weight may not have been evident at the time of parental report.'

Comment 3: Parents used subjective methods such as clothing size comparisons or visual assessments, they may have unintentionally underestimated their child's weight. It is therefore recommended that professionals consider objective weight measurement, using weighing scales data, especially for parents of young children.
Response 3: Yes we agree – within the 'limitations section, we have recognised this 'Child weight status was not independently measured. However, as parents typically underestimate their child's weight status, it is reasonable to assume that in most cases, a parents' concern regarding their child's weight was justified.' In the discussion, we have also recognised that the true proportion of children living with overweight or obesity in this cohort is likely higher than reported by parents. We have also now added a sentence to the' key messages' section regarding objective measures - 'Offering or sign-posting to child weight management support when a parent engages in a lifestyle weight management programme, could provide a valuable opportunity to augment positive indirect effects on the child and the opportunity to take objective measures of possible adiposity.'

Reviewer 2 - Dr. Sarthak Das, All India Institute of Medical Sciences - Deoghar

Conceptual & Methodological
Comment 1: Clarify the mixed-methods design and explicitly describe how quantitative and qualitative findings were integrated.
Response 1: In the abstract, we have clarified the mixed-methods design by adding to the methods section: 'In this mixed methods study,….' We have also added to the final sentence of the abstract methods 'qualitative' telephone interview analyses….
In the methods section, we have added a sentence at the start of the 'Study Design' section to state: 'This was a mixed methods study including quantitative analyses of a cross-sectional survey and qualitative analyses of telephone interview data and free text survey responses.' We also describe the integration of quantitative and qualitative findings further 'The themes identified helped contextualise aspects of the survey data.'

Comment 2: Acknowledge self-selection bias and limited generalisability due to recruitment through Slimming World and the predominantly female sample.
Response 2: In the UK, Slimming World is the largest adult commercial weight management programme and is embedded within National Health Service (NHS) referral pathways such as via the NHS digital weight management programme and in some areas, via dedicated GP referral pathways. Adult behavioural weight management services tend to include a predominantly female cohort (https://doi.org/10.3399/bjgp16X684337). Therefore, the findings of this study do have reasonable generalisability for the cohort of interest (i.e. adults attending a weight management programme) given most of these adults are women and Slimming World referrals are embedded within NHS referral pathways. We have added a sentence to the limitations section regarding this: 'However, as

most adults accessing behavioural adult weight management programmes in the UK are female, the findings of this study do have reasonable generalisability for this cohort of interest. Recruitment into the study was via a single adult weight management provider, which may introduce sampling bias. However, Slimming World is the largest commercial provider in the UK and is accessed both through NHS referral pathways and on a fee-paying basis, increasing the likelihood that the sample reflects a broad range of socioeconomic backgrounds.

Comment 3: Highlight the absence of objective child outcome measures and reliance on parent-reported diet, physical activity, and weight changes.
Response 3: We have highlighted this as a limitation in the 'Discussion-Limitations' section as follows: 'Dietary, physical activity and weight changes relied on self-report data from parents, which may be subject to recall bias.' We also have included the following 'Child weight status was not independently measured. However, as parents typically underestimate their child's weight status, it is reasonable to assume that in most cases, a parents' concern regarding their child's weight was justified'.

Comment 4: Report effect sizes and consider multivariable regression analyses; address multiple testing concerns.
Response 4: The quantitative analysis is intended to be descriptive rather than to explore causal relationships or formally test hypotheses. As such we have reported percentages in each category in Tables 1 and 3, and feel that regression analysis (and hence odds ratios/effect sizes) would risk overinterpreting the data, as there was limited diversity in the characteristics of survey respondents and limited data on relevant confounders.

We have provided p-values based on Chi-squared tests of independence as an indication of where patterns are not attributable solely to random variation, rather than as a formal hypothesis test, and throughout the paper, we do not draw conclusions based solely on p-values or use the term 'significant'.

We have made the following edits to clarify this in the Analysis section of Methods:
'Due to the nature of the survey, the quantitative analysis was descriptive. Survey data summarising motivating factors for the parent to address their own weight were presented as cross-tabulations by the parent's weight category (overweight, obesity 1, obesity 2 or obesity 3). Survey data related to parental concern about a child's weight were also presented as cross-tabulations by the parent's weight category, IMD quintile and length of SW membership. To aid interpretation, we report p-values for chi-squared tests of independence to identify those patterns that may or may not be attributable to random variation. However, these should be treated as indicative only, rather than as formal hypothesis tests.'

Comment 5: Clarify the cross-sectional nature of the study and potential confounding due to the COVID-19 period.
Response 5: We have added to the methods section:' 'This was a mixed methods study including quantitative analyses of a cross-sectional survey and qualitative analyses of telephone interview data and free text survey responses.'
In the limitations section we have added: 'Data collection occurred during the COVID-19 pandemic and associated confounding factors may have influenced parents' responses.'

Comment 6: Reframe pharmacological comparisons (e.g., GLP-1 agonists) as hypothesis-generating rather than evidence-based conclusions.
Response 6: We have edited the sentence within our discussion to make it clear that this is a hypothesis: 'The collateral effects reported by some parents on their children from lifestyle adult weight management programmes warrants further exploration with objective measures. This is particularly important given the rising popularity of pharmacological obesity treatments, which we

hypothesise may offer limited indirect benefits to children if given in isolation, without accompanying behavioural change interventions.'

COMMENTS (Interpretation & Depth)
Comment 7: Avoid overinterpretation of collateral benefits and emphasise heterogeneity of observed effects.
Response 7: We have emphasised the need for future work to include objective measures in the 'Key Messages' section. In the discussion (main findings), we have improved clarity with regards to the proportion of parents reporting collateral benefits on their child, to ensure that it is clear that this wasn't observed by all parents.

Comment 8: Include a brief gender-focused discussion highlighting the absence of paternal perspectives.
Response 8: In the discussion (limitations section) we state that 'Most parents included in this study were female, therefore paternal perspectives are not fully represented.' We have also highlighted that most adults accessing behavioural adult weight management programmes in the UK are female, therefore the findings of this study have reasonable generalisability for this cohort of interest.

Comment 9: Explicitly acknowledge the absence of the child's voice as a limitation.
Response 9: In the limitations section, we have added a sentence as follows: 'Finally, the study focuses on parental perspectives only, given that NICE advises families or carers to take main responsibility for behavioural changes in children and young people, especially children under 12. However, further studies may wish to explore the child's perspective'.

Comment 10: Expand interpretation of IMD findings and socioeconomic implications.
Response 10: In table 3, we report whether socio-economic deprivation is associated with parental concern about a child's weight. No association was observed between parental concern and IMD quintile. However, it's important to note that this study focuses on parents living with overweight or obesity (rather than parents of any weight status) and hence these children are already at increased risk of overweight or obesity. A separate study (https://doi.org/10.1371/journal.pone.0043503) reported that parental weight status appears to be the most significant independent predictor of childhood obesity across SES groups. This may partly explain why we don't observe differences in parental concern across SES groups, as the elevated baseline risk associated with parental overweight or obesity could attenuate any observable differences in parental concern across IMD quintiles. Furthermore, as parents from more deprived areas are more likely to underestimate a child's weight compared to those from more affluent areas (https://doi.org/10.3399/bjgp15X684385), this may also contribute towards the lack of observable difference across SES groups when parental report rather than objective measures are used. As this aspect wasn't the main focus of the study, we haven't expanded on this within the discussion.

QUALITATIVE METHODOLOGY
Comment 11: Add a reflexivity statement describing the interviewer's background and potential influence on data collection.
Response 11: We have added a statement within the limitations section as follows: 'Regarding reflexivity in the qualitative aspects of the study, it is recognised that RMs experience both as a General Practitioner and parent had potential to influence data collection, but open-ended questions helped mitigate this. Furthermore, the secondary male data analyst (AS) did not have a prior interest in the research topic other than that of methodologist and served to counter the potential for implicit bias in the analysis of interview data.'

Comment 12: Strengthen reporting of qualitative rigour by describing double coding, audit trail, and

adherence to COREQ standards.
Response 12: We have added further detail to the methods section to strengthen the reporting of qualitative rigour and also to the discussion related to interviewer reflexivity. We have included the COREQ checklist in our uploads to ScholarOne.

PRESENTATION & STRUCTURE
Comment 13: Simplify dense tables and highlight statistically meaningful findings more clearly.
Response 13: We have simplified the headings in Table 1. The use of 'statistically significant' is not recommended good practice, and is inappropriate for this study which is descriptive in nature. While we have included p-values to help identify patterns that may or may not be attributable to random variation, we have added a warning about the interpretation of these p-values to the Analysis section of the Methods (see response to comment 4).

Comment 14: Improve narrative explanation of figures within the Results section.
Response 14: We have edited the text within the Results section to improve clarity of wording associated with Figure 1.

Comment 15: Refine language to remove minor grammatical inconsistencies and improve conciseness.
Response 15: We have made edits to the manuscript to address this (For example, in the discussion, we have improved conciseness in relation to comparisons with NCMP data).

ETHICAL & CONCEPTUAL CONSIDERATIONS

Comment 16: Ensure consistent use of person-first and non-stigmatising language throughout the manuscript.
Response 16: We have edited 'children with overweight/obesity' to 'children living with overweight or obesity' in the 'limitations' section. We have edited 'children at higher risk of obesity' to 'children at higher risk of developing diabetes' in the discussion.

Comment 17: Acknowledge potential unintended negative collateral effects, such as transmission of dieting behaviours or food restriction to children.
Response 17: This is not something we specifically explored within our study, but in the limitations section we do discuss this. Within the discussion (contextualisation and implications of findings), we do also discuss other studies which have reported utilisation of weight control practices (both healthy and unhealthy) in children, where parents participated in an adult weight management programme.

KEY RECOMMENDATIONS BEFORE PUBLICATION
Comment 18: Strengthen the limitations section, particularly addressing bias, self-reporting, and lack of causal inference.
Response 18: We have strengthened the limitations section, specifically referring to sampling and re-call bias. We have highlighted and discussed self-reported data. We have also discussed reflexivity.

Comment 19: Temper conclusions regarding collateral benefits.
Response 19: In the Discussion section, we have added to the following sentence, highlighting that our findings need further exploration with objective measures: 'The collateral effects reported by parents on their children from lifestyle adult weight management programmes warrants further exploration with objective measures.'

Comment 20: Enhance qualitative methodological transparency.
Response 20: We have edited the methods section to add further detail.

Comment 21: Reduce speculative discussion not directly supported by data.
Response 21: We have made some minor edits to the discussion.

Comment 22: Consider additional adjusted statistical analyses if data permit.
Response 22: As noted in our earlier response to comment 4, this was a descriptive study with limited data and so we do not feel that adjusted regression analyses are warranted here. We have made some edits to the text to clarify this; please see earlier response.

VERSION 2 – REVIEW

**REVIEWER NAME**	Das, Sarthak
**REVIEWER AFFILIATION**	All India Institute of Medical Sciences - Deoghar, Pediatrics
**REVIEWER CONFLICT OF INTEREST**	No Conflict of Interest
**AI DISCLOSURE**	No, I have not used AI
**DATE REVIEW RETURNED**	17-Apr-2026

**GENERAL COMMENTS**	Reviewer Comments (Second Review – Minor Revision)<br>Overall Assessment<br>The manuscript addresses an important and underexplored area—collateral effects of parental participation in weight management programmes on child health behaviors. The mixed-methods approach is appropriate and the topic has clinical and public health relevance. However, several methodological, analytical, and interpretative concerns remain.<br>Major Comments<br>1. Study design and causality<br>The study is cross-sectional with self-reported outcomes, yet conclusions imply causal effects. Please reframe conclusions to reflect associations, not causation.<br>2. Parent-reported child outcomes<br>All child outcomes are subjective and parent-reported, introducing bias. Avoid overinterpreting behavioral changes as clinical improvements.<br>3. Lack of objective data<br>No objective child BMI or measurements were included. The assumption that parental concern reflects true obesity should be tempered.<br>4. Sampling bias<br>The sample is predominantly female and from a single programme, limiting generalizability.<br>5. Statistical concerns<br>Inconsistency between descriptive analysis and use of p-values. Clarify statistical approach.<br>6. Mixed-methods integration<br>Qualitative and quantitative findings are not well integrated. Consider triangulation.<br>7. Interpretation of benefits<br>A substantial proportion reported no change in children. Avoid overly optimistic conclusions.<br>8. COVID-19 confounding<br>Pandemic-related factors may have influenced results and should be emphasized.<br>Minor Comments<br>1. Abstract<br>Use non-causal language and include key limitations.<br>2. Methods clarity<br>Provide response rate, missing data handling, and sampling

	rationale.<br>3. Tables<br>Clarify denominators and highlight no-change proportions.<br>4. Terminology<br>Use consistent and non-stigmatizing language.<br>5. Discussion<br>Reduce speculative statements about pharmacological treatments.<br>6. Patient involvement<br>Briefly discuss absence of patient/public involvement.<br>Recommendation<br>Minor Review

VERSION 2 – AUTHOR RESPONSE

Comment 1: The manuscript addresses an important and underexplored area—collateral effects of parental participation in weight management programmes on child health behaviours. The mixed-methods approach is appropriate and the topic has clinical and public health relevance. However, several methodological, analytical, and interpretative concerns remain.
Response 1: Thank you for taking the time to review this manuscript and your recognition of this important area of research with both clinical and public health relevance. Please find our responses to the remaining comments below.

Comment 2: Study design and causality
The study is cross-sectional with self-reported outcomes, yet conclusions imply causal effects. Please reframe conclusions to reflect associations, not causation.
Response 2: In the conclusion we are clear that the dietary and physical activity lifestyle changes described for the children were those reported by the parent rather than using objective measures. In the discussion, we outline the limitations associated with self-report data. However, in response to this comment, we have further edited the manuscript from 'some reported that their child had already indirectly benefited' to 'some reported that their child's diet and physical activity levels had improved.' We have also added a sentence to the conclusion stating 'Future research should explore these subjective parental observations using objective measures.'

Comment 3: Parent-reported child outcomes
All child outcomes are subjective and parent-reported, introducing bias. Avoid overinterpreting behavioural changes as clinical improvements.
Response 3: In the conclusion, we are clear that the dietary and physical activity lifestyle changes described for the children were those observed by the parent rather than objective measures but we have edited the conclusion further as described in the previous response. In the discussion, we clearly describe the limitations in relation to child outcomes being parent-reported.

Comment 4: Lack of objective data
No objective child BMI or measurements were included. The assumption that parental concern reflects true obesity should be tempered.
Response 4: We have responded to the previous peer review comment re absence of objective child outcome measures and reliance on parent-reported measures, including in the discussion-limitations section 'Dietary, physical activity and weight changes relied on self-report data from parents, which may be subject to recall bias.' We had also included the following 'Child weight status was not independently measured.' However, as it is well established within the literature that parents typically underestimate their child's weight status, we had also included the following: 'However, as parents typically underestimate their child's weight status, it is reasonable to assume that in most cases, a parents' concern regarding their child's weight was justified'. In Figure 1, we make it very clear that we are reporting parental concern about a child's weight i.e. 'proportion of parents worried child is living

with overweight' rather than objective measures of weight, such as child BMI z-scores. In response to comment 2 of this peer review, we have also added a sentence to the conclusion regarding the need for future research using objective measures.

Comment 5: Sampling bias - The sample is predominantly female and from a single programme, limiting generalizability.
Response 5: As outlined in the response to the previous peer review, adult behavioural weight management services in the UK typically include a predominantly female cohort (https://doi.org/10.3399/bjgp16X684337). Therefore, the findings of this study do have reasonable generalisability for this cohort of interest. Slimming World is the largest commercial provider in the UK for adult lifestyle weight management services and is accessed both through NHS referral pathways and on a fee-paying basis. It also has a predominance of female membership ('94% of the membership is female': https://doi.org/10.1016/j.nutos.2023.07.003). In the discussion we recognise this 'Most parents included in this study were female, therefore paternal perspectives are not fully represented. However, as most adults accessing behavioural adult weight management programmes in the UK are female, the findings of this study have reasonable generalisability for this cohort of interest [references 35 and 36].'

We also have already specifically discussed sampling bias in the discussion 'Recruitment into the study was via a single adult weight management provider, which may introduce sampling bias. However, Slimming World is the largest commercial provider in the UK and is accessed both through NHS referral pathways and on a fee-paying basis, increasing the likelihood that the sample reflects a broad range of socioeconomic backgrounds.'

Comment 6: Statistical concerns
Inconsistency between descriptive analysis and use of p-values. Clarify statistical approach.
Response 6: In response to previous comments, we emphasised the predominately descriptive nature of the study. The p-values are provided not to highlight 'important results' but more to avoid too much attention on patterns that may appear important but that due to the sample size involved are due to random variation.

We have now made additional edits to clarify this and ensure consistency across the manuscript. In addition, we have removed the p-values from the main text. While we have kept them in the tables for the purpose of interpretation, we have included a caveat as a footnote.

Comment 7: Mixed-methods integration - Qualitative and quantitative findings are not well integrated. Consider triangulation.
Response 7: With regard to data triangulation, we were looking to expand our understanding of the findings of the survey participation and enhance the credibility of the findings. The interviews were conducted post-hoc and the topics addressed were informed by the survey responses. In so doing, we reiterate that the mixed-methods approach employed served to better understand the rationale for survey responses and the provision of the greater context that is described in the Results section and appraised in the context of limitations in the Discussion.

Comment 8: Interpretation of benefits - A substantial proportion reported no change in children. Avoid overly optimistic conclusions.
Response 8: We have included a sentence in the results section stating: 'Forty-six percent of parents did not observe any changes in their children's diet, physical activity or weight.' The conclusion has been edited in response to other comments.

Comment 9: COVID-19 confounding
Pandemic-related factors may have influenced results and should be emphasized.

Response 9: In response to the previous peer review, we recognised this as a limitation, reporting in the discussion: ‘Data collection occurred during the COVID-19 pandemic and associated confounding factors may have influenced parents’ responses.’

Comment 10: Abstract -- Use non-causal language and include key limitations.
Response 10: Limitations are clearly outlined within the manuscript, but we have added to the abstract methods section to make it clear that parent-reported measures (rather than objective measures) were collected: ‘This included parent-reported measures of BMI and their child’s weight status, physical activity and dietary behaviours.’ In the results section of the abstract, we outline what parents reported, since they had taken steps to address their own weight. We make it clear that this is what parents reported unverified by objective measures

Comment 11: Methods clarity
Provide response rate, missing data handling, and sampling rationale.
Response 11: As the survey was advertised on the members-only section of the Slimming World website, it is not possible to provide a response rate. We have added a sentence to the limitations section as follows: ‘As the survey was advertised on the Slimming World members-only section of their website, it is not possible to report a response rate.’
Where data were missing, this is clearly reported in the results section (e.g. footnote in Table 1 and 3- one parent did not provide a BMI). We have already discussed re sampling rationale in the discussion ‘Slimming World is the largest commercial provider in the UK and is accessed both through NHS referral pathways and on a fee-paying basis, increasing the likelihood that the sample reflects a broad range of socioeconomic backgrounds.’

Comment 12: Tables - Clarify denominators and highlight no-change proportions.
Response 12: We have already included denominators within the tables (e.g. Table 1- n=395 as BMI missing for one parent). We have added a sentence to the results: ‘Forty-six percent of parents did not observe any changes in their child’s diet, physical activity or weight.’

Comment 13: Terminology - Use consistent and non-stigmatizing language.
Response 13: We have reviewed the manuscript and do not feel that stigmatizing language has been used. Actual parental quotes have been reported verbatim as told to us in interviews. We do not feel it appropriate to change the participants’ voices.

Comment 14: Discussion - Reduce speculative statements about pharmacological treatments.
Response 14: Given the advances in the management of obesity in recent years, with new pharmacological treatments available, we feel that it is warranted and appropriate to include discussion related to these within the manuscript. Within the discussion, we only include one sentence related to pharmacological treatments and make it clear within this sentence that we are hypothesising rather than drawing any conclusive statements. If pharmacological support is given alone to the parent, and the parent doesn’t make any behavioural changes in conjunction, then we feel that it is reasonable to hypothesise that they may not observe any changes in their child’s lifestyle behaviours (diet, physical activity levels). In the ‘key messages’, we raise this as a future research question.

Comment 15: Patient involvement
Briefly discuss absence of patient/public involvement.
Response 15: The manuscript already includes a section re ‘Patient and Public Involvement’ stating ‘Patients or the public were not involved in the design or conduct, or reporting, or dissemination plans of our research.’
